# Opioid Analgesia and Opioid-Induced Adverse Effects: A Review

**DOI:** 10.3390/ph14111091

**Published:** 2021-10-27

**Authors:** Alok K. Paul, Craig M. Smith, Mohammed Rahmatullah, Veeranoot Nissapatorn, Polrat Wilairatana, Mariana Spetea, Nuri Gueven, Nikolas Dietis

**Affiliations:** 1 School of Pharmacy and Pharmacology, University of Tasmania, Hobart, TAS 7001, Australia; nuri.guven@utas.edu.au; 2 School of Medicine, Institute for Mental and Physical Health and Clinical Translation, Deakin University, Geelong, VIC 3216, Australia; craig.smith@deakin.edu.au; 3 Department of Biotechnology & Genetic Engineering, University of Development Alternative, Dhanmondi, Dhaka 1207, Bangladesh; rahamatm@hotmail.com; 4 School of Allied Health Sciences, World Union for Herbal Drug Discovery (WUHeDD) and Research Excellence Center for Innovation and Health Products (RECIHP), Walailak University, Nakhon Si Thammarat 80160, Thailand; nissapat@gmail.com; 5 Department of Clinical Tropical Medicine, Faculty of Tropical Medicine, Mahidol University, Bangkok 10400, Thailand; 6 Department of Pharmaceutical Chemistry, Institute of Pharmacy and Center for Molecular Biosciences (CMBI), University of Innsbruck, Innrain 80–82, 6020 Innsbruck, Austria; mariana.spetea@uibk.ac.at; 7 Medical School, University of Cyprus, Nicosia 1678, Cyprus; dietis.nikolas@ucy.ac.cy

**Keywords:** opioids, morphine, analgesia, chronic pain, behaviour, adverse effects, tolerance

## Abstract

Opioids are widely used as therapeutic agents against moderate to severe acute and chronic pain. Still, these classes of analgesic drugs have many potential limitations as they induce analgesic tolerance, addiction and numerous behavioural adverse effects that often result in patient non-compliance. As opium and opioids have been traditionally used as painkillers, the exact mechanisms of their adverse reactions over repeated use are multifactorial and not fully understood. Older adults suffer from cancer and non-cancer chronic pain more than younger adults, due to the physiological changes related to ageing and their reduced metabolic capabilities and thus show an increased number of adverse reactions to opioid drugs. All clinically used opioids are μ-opioid receptor agonists, and the major adverse effects are directly or potentially connected to this receptor. Multifunctional opioid ligands or peripherally restricted opioids may elicit fewer adverse effects, as shown in preclinical studies, but these results need reproducibility from further extensive clinical trials. The current review aims to overview various mechanisms involved in the adverse effects induced by opioids, to provide a better understanding of the underlying pathophysiology and, ultimately, to help develop an effective therapeutic strategy to better manage pain.

## 1. Introduction

### 1.1. Pain

According to the International Association for the Study of Pain (IASP), pain is defined as an unpleasant sensory and emotional experience associated with actual potential tissue damage or described in terms of such damage [1]. Pain can be categorised as nociceptive, neuropathic or nociplastic pain (a combination of both that cannot be entirely explained as nociceptive or neuropathic). Nociceptive pain is generated as a warning signal transmitted to the brain about the possible damage of a non-neural tissue [1,2]. In contrast, neuropathic pain usually results from damage to neural tissue by a disease, toxin or infection [1,3]. The third type of pain, the nociplastic pain, is a complex pain, not completely defined but probably caused by an alteration of neurons’ pain response and an increased sensitivity of the central nervous system (CNS). Nociceplastic pain generally presents over 3 months of duration with symptoms such as hyperalgesia and regional pain sensations and is commonly observed in patients with cancer and other long-term chronic disorders [4,5,6].

The opioid system is a physiological control system that modulates pain, emotions, immune defence and various other physiological responses. The opioid system involves the communication and coordination of a significant number of endogenous opioid peptides and several types of opioid receptors in the CNS and peripheral nervous system. This system also significantly modulates numerous sensory, emotional, motivational and cognitive functions, as well as addictive behaviours [7,8,9]. It is also involved in other physiological functions, including responses to stress, respiration, gastrointestinal transit, endocrine and immune functions [10]. These responses are orchestrated by opioid ligands that bind to specific opioid receptors to induce analgesia and behavioural effects in vivo. Therefore, to understand the pharmacological effects of specific opioids, it is first essential to clarify the specific roles of each opioid receptor type.

### 1.2. Opioid Receptors

The presence of opioid receptors was first proposed in 1954 [11]. However, the first evidence of the multiplicity of opioid receptors was only described in 1976 [12]. According to the International Union of Basic and Clinical Pharmacology (IUPHAR) and the British Pharmacological Society (BPS) joint *IUPHAR/BPS Guide to Pharmacology*, opioid receptors are classified into μ (Mu: MOP), δ (delta: DOP) and κ (kappa: KOP) receptors, as well as the non-classical nociception (NOP) receptor [13] (Table 1).

Opioid receptors belong to the family of seven-transmembrane helical G protein-coupled receptors (GPCRs) and share about 60% homology in the amino acid composition. These receptors display an extracellular N-terminus and an intracellular C-terminus and are coupled with heterotrimeric Gi/Go proteins [15,16,17]. Opioid ligands bind to opioid receptors by establishing ligand–receptor interactions in the binding pockets of the receptor, which are situated in the transmembrane helices. The binding pocket of opioid receptors can be divided into two distinct regions; the lower part (intracellular side) of the receptor is highly conserved for opioids (non-specific ‘message’ region), and the higher part of the pocket (extracellular side) contains divergent residues that confer selectivity (‘address’ region) to opioid receptor types; binding also depends on the type of the opioid ligand [18,19]. In 2012, the first molecular structures of all four opioid receptors were described in several reports [18,20,21,22].

Although all types of opioid receptor types modulate analgesia, the MOP receptor is thought to be dominant for its pain-relieving effects [23,24,25,26]. The major limitation of targeting the MOP receptor for analgesia is that it is also responsible for the induction of tolerance [27] and other undesirable adverse effects including addiction [28,29], dependence, respiratory depression [30] and constipation [31]. The MOP receptor is expressed in the brain, spinal cord and elsewhere in the body, and the adverse effects are relevant to its site of activation [28]. For example, in the gut, MOP receptor activation can cause constipation. However, the most important activation site is in the brain, as the MOP receptor drives hedonic reward, reinforcing, addictive, tolerance, dependence and withdrawal symptoms [32]. It is presumed that peripherally restricted MOP receptor agonists (that do not pass the blood–brain barrier) mediate local analgesia (effective against inflammatory or neuropathic pain) with reduced centrally mediated adverse effects [28]. MOP receptor-related adverse events are of great clinical concern and justify the characterisation of other opioid receptor types as suitable drug targets to induce analgesia. Unfortunately, the other three opioid receptor types (DOP, KOR and NOP receptors) do not have the same efficacy in mediating analgesia compared to the MOP receptor. DOP receptor agonists are generally less effective to treat acute thermal pain compared to inflammatory [33,34,35], neuropathic [36,37] and cancer-associated bone pain [38]. SNC80 and deltorphin II, two selective DOP receptor agonists, show significant anti-hyperalgesic effects, but these agonists are less potent or less efficacious in inducing thermal antinociceptive effects [34]. In addition, the use of DOP receptor agonists is limited, since DOP receptor-induced analgesia appears to require the presence of a pro-inflammatory state [39,40]. While DOP receptor agonists only produce moderate analgesia in non-human primates [41,42,43], despite being effective in rodent models of chronic pain [44], they are associated with convulsions in mice [45] and non-human primates [41,42,43]. Additionally, KOP receptor agonists are reported to reduce visceral [46,47], inflammatory [48,49] and neuropathic pain [50,51], but they also produce CNS-associated adverse events (i.e., dysphoria, psychotomimesis) [52,53,54]. While selective KOP and DOP receptor agonists lack some of the MOP receptor-mediated liabilities, such as constipation, respiratory depression and addiction, they display a side effect profile of their own [55]. Several NOP receptor agonists are reported to have antinociceptive effects in rodent [56,57] and primate models [58,59,60] and are associated with a reduced risk for abuse [61]. However, systemic administration of NOP agonists did not produce spinal analgesia in rodents [62,63], while showing efficacy after intrathecal administration in primates and rodent models of neuropathic pain [57,58,61,64]. Overall, MOP receptor agonists, despite their adverse effects, remain the most efficacious drugs in providing pain relief and are thus widely used in the clinic [26,65].

In the investigation on the role of specific opioid receptors and their ligands in pain modulation, antinociceptive tolerance and adverse behavioural effects, the generation of knockout animals has provided significant knowledge on the in vivo physiological role of the opioid system. For example, in MOP receptor knockout mice, MOP receptor agonist-induced antinociception and their associated side effects (e.g., hyperlocomotion, respiratory depression, inhibition of gastrointestinal tract transit, reward and withdrawal effects) were effectively abolished [23,66,67]. At the same time, morphine efficiently induced analgesia in DOP [68] and KOP [69] receptor knockout mice, albeit with reduced adverse effects (i.e., tolerance and withdrawal response). Similarly, KOP receptor agonists are also reported to induce analgesia in MOP [70] and DOP [68] receptor knockout mice, while predictably, in KOP receptor knockout animals, this effect was not observed [69]. However, DOP receptor agonists show only reduced levels of analgesia in DOP receptor knockout mice [68], although a mixed effect (decreased/maintained) on analgesia was observed in MOP receptor knockout mice [70,71].

### 1.3. Endogenous Opioid Ligands

Opioid ligands are from both endogenous and exogenous origins. Evidence of the existence of endogenous ligands for the opioid receptors was obtained in the 1970s, and the structures of [Leu]- and [Met]-enkephalin were reported in 1975 [72]. Endogenous opioid peptides are found in the CNS and peripheral nervous system and in the gastrointestinal tract [73]. These peptides are derived from the four different precursors pro-enkephalin, pro-dynorphin, pro-opiomelanocortin and prepro-nociceptin [15,74,75,76]. Pro-enkephalin contains two 267 amino acid polypeptides [77], and mainly produces the pentapeptides [Leu]- and [Met]-enkephalins [78,79] with selectivity for MOP and DOP receptors. Dynorphins are mainly big dynorphin, dynorphin A, dynorphin B and α-neo-endorphin and interact mainly with the KOP receptor [80,81]. Endorphins are derived from pro-opiomelanocortin [82] and are expressed as α-, β- and γ-endorphins [83]. While endorphins activate the MOP receptor, the prepro-nociceptin-derived neuropeptide nociceptin/orphanin FQ binds to the NOP receptor [7]. Endogenous opioids affect a multitude of physiological functions, such as pain modulation and analgesia, stress and emotional responses, tolerance and dependence, learning and memory, addiction, sexual activity and control of hormone levels, neurological disorders, eating and drinking behaviour, gastrointestinal, renal and hepatic functions, cardiovascular responses, respiration, thermoregulation and immunological responses [84,85].

### 1.4. Exogenous Opioid Ligands

Over more than 8000 years, the poppy plant (*Papaver somniferum*) and the opioids derived from it have been used for pain relief. In a Sumerian ideogram, the poppy plant was known as a “plant of joy” [85]. Crude opium admixtures were widely used in different British and German medicines in the 16th century, and effects like pain tolerance and physical dependence on opioids were noted at this time. In 1805, Friedrich Sertürner isolated morphium (morphine) and named it after the Greek god Morpheus (the “God of sleep and dreams”). Within two decades after the initial isolation of morphine, commercial production of morphine started, and morphine became available on the European market. Subsequently, after the invention of hypodermic syringes in the middle of the 19th century, morphine was injected systematically into painful areas [85].

Currently, different alkaloids extracted from the poppy plant (*Papaver somniferum*), including opium, morphine and codeine, are still used for pain relief, mood disorders and palliative care. In addition, several semi-synthetic and synthetic opioids, such as buprenorphine, dextropropoxyphene, hydromorphone, oxycodone, pethidine, fentanyl, methadone, tapentadol and tramadol, are widely used in patients that suffer from surgical or chronic pain [86].

### 1.5. Opioid Receptor-Mediated Signalling Pathways

**Figure 1:**
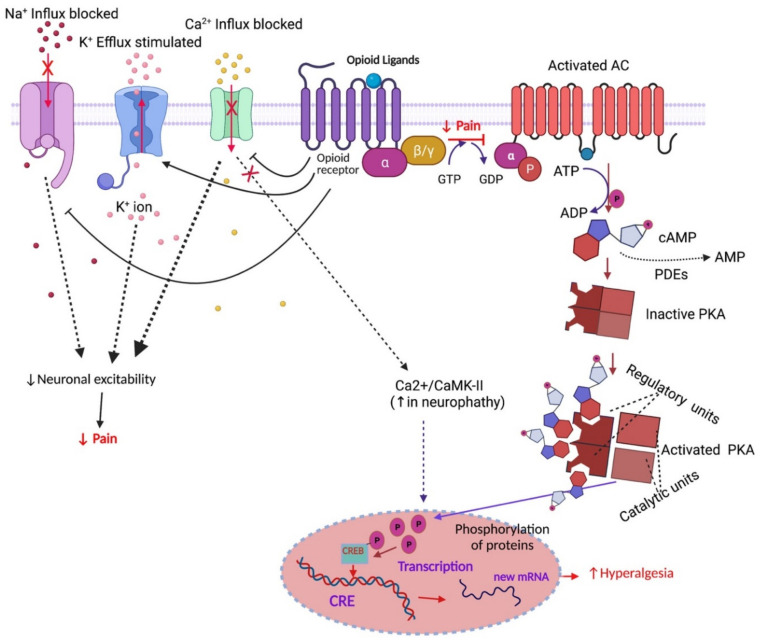
Effects of opioid agonists on pain and signal transduction. Abbreviations: ATP, adenosine triphosphate; ADP, adenosine diphosphate; GABA, gamma-aminobutyric acid; AMP, adenosine monophosphate; cAMP, cyclic adenosine monophosphate; PKA, protein kinase A; CRE, cAMP-responsive element; CREB, cAMP-responsive element binding protein; GTP, Guanosine triphosphate; GDP, Guanosine diphosphate; PDEs, Cyclic nucleotide phosphodiesterases; Ca2+/CaMK-II, Ca2+/calmodulin-dependent protein kinase II. Symbols: solid arrow, strong activation; dashed arrow, moderate activation; solid T-shaped line, strong inhibition; dashed T-shaped line, moderate inhibition, line with ×, blocking; upward arrow, increased effect; downward arrow, decreased effect. The figure was made with www.biorender.com (accessed on 19 October 2021).

Once an agonist binds to a GPCR, it activates a G-protein (a heterotrimeric protein composed of three different subunits (α, β and γ)). The α subunit (which can be Gs, Gi, Go, Gq) exchanges a bound guanosine diphosphate (GDP) for a guanosine triphosphate (GTP), which activates the subunit and dissociates it from the βγ subunits. The α subunit goes on to activate/inhibit different downstream signal transduction pathways. For example, it inhibits (Giα) or activates (Gsα) adenylyl cyclase, which leads to the activation or inhibition of the production of cyclic adenosine monophosphate (cAMP) from ATP [87,88]. This activation modulates voltage-gated calcium, sodium and potassium channels [89,90] and cellular levels of cAMP or the activity of protein kinase A (PKA) [91] (Figure 1). Activated PKA translocates to the nucleus, where it induces the phosphorylation of cAMP response element-binding protein (CREB) (Figure 1). Phosphorylated CREB facilitates the desired gene expression levels as the promoters contain cAMP response elements (CRE) and control different cellular functions [92] (Figure 1). Increased activity of CREB has been observed in cancer cells, chronic inflammatory or neuropathic pain conditions, and blocking CREB can inhibit cell proliferation, differentiation and survival, as well as peripheral neuropathy [93,94,95,96] (Figure 1). However, another study showed that CREB has a dual role in cell proliferation (stimulation or inhibition), and its function depends on the pathway of activation [97]. For example, cAMP-activated PKA-phosphorylated CREB stimulates mitosis (cell proliferation), but growth factor-activated CREB inhibits mitosis [97]. Opioid peptides activate membrane-bound receptors on sensory nerve fibres and produce acute analgesia by reducing the excitability of sensory neurons. Activation of the MOP receptor by both endogenous and exogenous opioids on post-synaptic neurons dissociates the G_α_ subunit from the G_βγ_ subunits of the G protein, which increases potassium (K^+^) conductance in neurons. The resulting efflux of K^+^ ions hyperpolarise the neuronal cells and reduces their excitability [98,99,100]. Opioids binding to opioid receptors also inhibits calcium (Ca^2+^) and sodium (Na^+^) ion influx, which reduces Ca^2+^- and Na^+^-induced depolarisation of neurons [65,95,101,102].

Different ligands can act through the same receptors but trigger distinct intracellular transduction pathways, and this phenomenon is termed biased agonism [103,104,105,106]. Biased agonism of the MOP receptor is nowadays investigated to avoid antinociceptive tolerance and other adverse effects, with a particular focus on the development of ligands with no β-arrestin-2 (β-arr2) recruitment [107,108,109]. Increased antinociceptive response and reduced adverse effects were observed for ligands with decreased β-arr2 recruitment or in β-arr2 knockout mice [109,110,111,112,113,114]. Biased agonists for the KOP receptor with reduced β-arr2 recruitment show antinociception and antipruritic effects, with fewer adverse effects than selective unbiased KOP receptor agonists [115,116]. Oliceridine, a MOP receptor biased ligand, displayed similar efficacy on MOP receptor binding, but showed a six-fold reduced efficacy on β-arr2 recruitment [117]. In the clinic, oliceridine was demonstrated to be an effective painkiller with a reduced side effect profile compared to morphine [117,118].

## 2. Opioid-Induced Adverse Effects

### 2.1. Analgesic Tolerance

The development of analgesic tolerance to opioids after repetitive administration is one of the major limitations for their chronic use in the clinic. Morphine is one of the most effective and widely prescribed drugs against severe pain [26,119]. However, long-term morphine treatment is discouraged in the clinic due to the risk of adverse effects, including analgesic tolerance [120,121]. Tolerance manifests as decreased drug efficacy following repeated administration [122]. Therefore, to maintain efficacy, dose increments are required, which in turn contribute to generating cellular desensitisation, tolerance, physical dependence and behavioural withdrawal symptoms. In addition, increased morphine dosing is frequently required due to amplified disease progression rather than analgesic tolerance [123].

The clinical management of analgesic tolerance involves opioid rotation and the combination of opioids with adjuvants [124,125,126,127]. Adjuvants, such as gabapentin, pregabalin, dexamethasone, naproxen, ibuprofen, carbamazepine, aspirin, venlafaxine and acetaminophen, are combined with opioid analgesics in patients that require long-term analgesic treatment [128,129]. Similarly, in preclinical studies, a combination of opioids and non-opioid adjuvants or combinations of opioid agonist and antagonist are used to prevent antinociceptive tolerance [130,131,132,133]. The activation of the opioid receptors leads to receptor phosphorylation by GPCR kinases, which promotes the interaction with β-arr [134,135]. Both phosphorylation and interaction with β-arr are required for subsequent receptor internalisation [134,135]. This internalised receptor can be proteolytically degraded. However, receptors can also be recycled in endosomes to be returned to the cell membrane [135,136]. This process is called receptor trafficking. In addition, de novo receptor synthesis ensures that new opioid receptors are produced and transported to the cell membrane via the trans-Golgi network [135]. Prolonged treatment with opioids increases the number of inactive (phosphorylated) receptors on the membrane, as well as the number of de novo synthesised receptors [135,136]. A clinical study on the use of opioids in cancer-related chronic pain showed that chronic administration of opioids induces increased methylation of the MOP receptor gene (OPRM1) on peripheral leucocytes and causes analgesic tolerance, but the article reported that a preclinical study on mice showed that targeted re-expression of the MOP receptor (by gene therapy) in cancer cells can reverse analgesic tolerance [137].

Specifically, chronic exposure to morphine leads to the selective recruitment of β-arr2 but not of β-arr1 [138]. In contrast to the interaction with β-arr1, which leads to receptor recycling, β-arr2 does not results in opioid receptor recycling but increases the number of inactive receptors on the cell membrane. This process is associated with insufficient analgesia [138]. Although the molecular mechanisms that lead to opioid tolerance are not entirely clear, both desensitisation and trafficking are assumed to be the key factors that lead to insufficient analgesia [107,135,138,139]. Although it is essential to delineate the exact molecular mechanisms resulting in opioid tolerance [138,140,141,142,143], it is also important to understand how chronic morphine dosing itself can influence analgesic tolerance and associated behavioural dependence [144,145].

The antinociceptive effects of morphine and other opioids in preclinical studies are commonly measured as central (brain and spinal cord) or peripheral antinociception [146,147]. The commonly used tail-flick test potentially measures spinal-mediated nociception, while the hot-plate assay largely measures supraspinal-mediated nociception [147,148]. Generally, one such antinociception test is performed in preclinical studies with repeated morphine treatment. As a result, the progression of antinociceptive tolerance measured by a single pain assay may be different when using another assay [145].

### 2.2. Addiction and Physical Dependence

**Figure 2:**
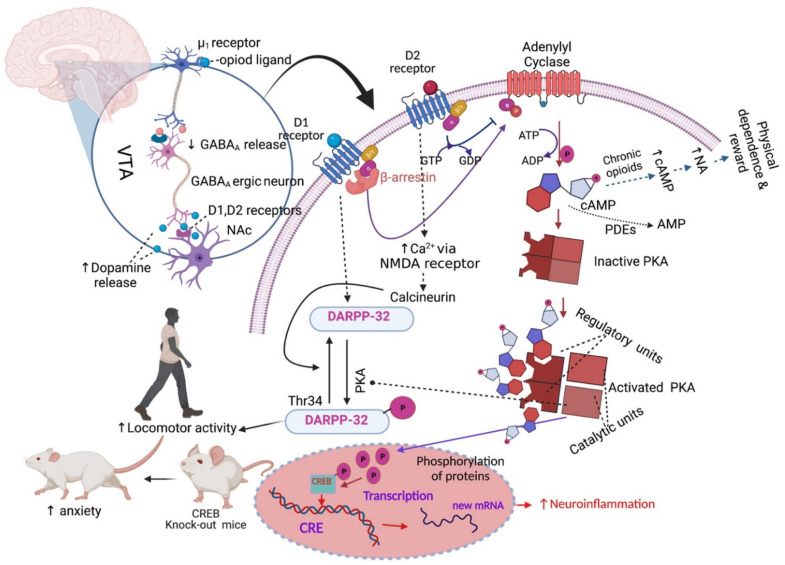
Effects of opioids on motor behaviour. Abbreviations: GABA, gamma-aminobutyric; cAMP, cyclic adenosine monophosphate; PKA, protein kinase A; CRE, cAMP-responsive element; CREB, cAMP-responsive element binding protein; D1, dopamine receptor-1; DARPP-32, dopamine- and cAMP-regulated phosphoprotein of 32 kDa; NMDA, *N*-methyl-*D*-aspartate receptor; PDEs, Cyclic nucleotide phosphodiesterases; NA, noradrenaline, VTA, ventral tegmental area; NAc, nucleus accumbens. Symbols: solid arrow, strong activation; dashed arrow, moderate activation; solid T-shaped line, strong inhibition; dashed T-shaped line, moderate inhibition, upward arrow, increased effect; downward arrow, decreased effect. The figure was made with www.biorender.com (accessed on 19 October 2021).

Apart from analgesic tolerance, long-term opioid treatment also causes behavioural adverse effects like physical dependence and addiction to these drugs. Physical dependence consists of craving for a drug either for pleasure or to avoid the occurrence of withdrawal symptoms following a reduction of the treatment dose or the intake of an opioid receptor antagonist [149,150]. Addiction indicates a loss of control of opioids use [150]. Physical dependence is associated with the upregulation of cAMP and noradrenergic signalling in the locus coeruleus (LC) neurons of the dorsal pontine tegmentum of the brainstem [151,152,153]. The molecular mechanism that initiates physical dependence and reward is associated with repeated opioid treatment [151,154,155]. Briefly, MOP receptor binding with opioids, like morphine, causes dopamine release by dopaminergic neurons in the VTA, VTA neurons transfer dopamine to the NAc, and this induces a pleasure feeling [154] (Figure 2). After chronic intake of opioids, a larger amount of opioids is required gradually to stimulate the VTA neurons and sustain the release of a similar amount of dopamine in the NAc. Thus, patients become dependent and tend to take more drugs to feel better [154]. The LC region of the brain that controls noradrenaline release is responsible for the dependence and reward processes [154].

Endogenous opioids bind to opioid receptors in neuronal cell bodies of the LC and stimulate adenylyl cyclase to convert ATP to cAMP, but acute opioid intake, e.g., of morphine or heroine, inhibits the conversion, and as a result, less cAMP is produced. As noradrenaline release is stimulated by cAMP, less noradrenaline is released in the LC [154]. Noradrenaline stimulates wakefulness, respiration, and several other processes. Repeated opioids intake causes desensitisation of the opioid receptors; thus, neuronal cells produce a similar amount of ATP and cAMP in the presence of a higher concentration of opioids in the LC [154]. Chronic morphine administration increases the levels of type I and VII of adenylyl cyclase, PKA subunits and several phosphoproteins (e.g., CREB) and results in the hyperactivation of the cAMP pathway [152] (Figure 2). Then, if the patient stops taking opioids, this causes a massive release of noradrenaline in the LC neurons, which causes anxiety, nervousness and muscle cramps [154]. Clinical guidelines for long-term opioid use propose a “start low and go slow” dosing regimen to prevent addiction, physical dependence, overdosing or abuse [120,156,157,158,159,160]. Therefore, clinical guidelines propose administering the smallest effective dose [161], rather than aiming for adequate long-term pain relief [162].

### 2.3. Constipation

**Figure 3:**
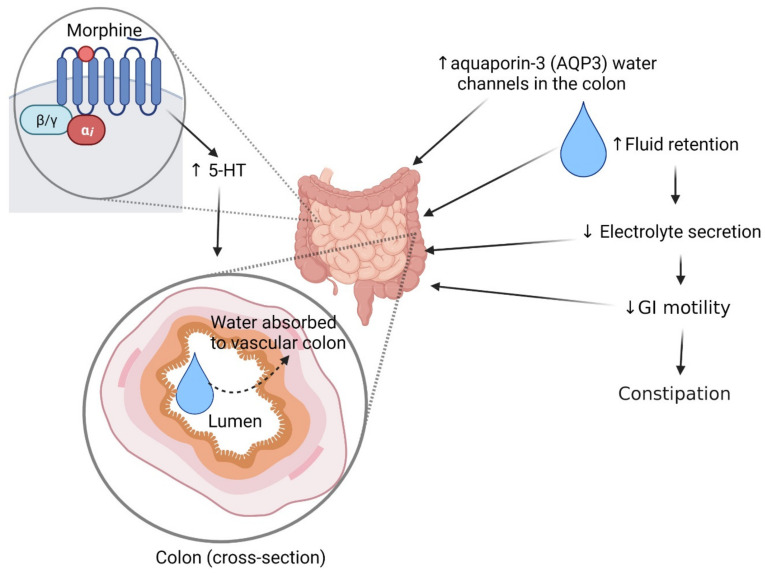
Effects of opioids in the gastrointestinal tract. Abbreviations: 5-HT, serotonin; GI, gastrointestinal. Symbols: solid arrow, strongly connected; dashed arrow, moderately connected; upward arrow (symbol), increased effect; downward arrow (symbol), decreased effect. The figure was made with www.biorender.com (accessed on 19 October 2021).

Constipation is a very common unwanted side effect of opioids and is caused by the activation of the MOP receptor in the enteric nervous system [163,164]. Opioids bind to MOP receptors in enteric neurons and delay gastrointestinal (GI) transit time, which also stimulates non-propulsive GI motility and pylorus and ileocecal sphincters [134]. Morphine treatment increases the expression of aquaporin-3 (AQP3) water channels in the colon by increased secretion of serotonin (5-HT), which increases water absorption from the luminal part to the vascular part of the colon [165] (Figure 3). As a result, constipation develops by increased fluid absorption from the large intestine along with less electrolyte secretion by the intestinal lumen [164] (Figure 3). In contrast, chronic morphine treatment does not produce tolerance to reduced GI motility in the lower GI tract, while it induces analgesic tolerance and leaves GI motility unaffected in the upper GI tract [166]. As a result, patients over a long-term opioid treatment continuously suffer from constipation. Constipation affects about 40% of patients with chronic oral opioid treatment, and therefore, different laxatives and non-medication approaches (e.g., fibrous diet, hydration) are used to provide comfort to the patients [149,167,168,169]. In addition, opioids combined with a low dose of opioid antagonists, such as naloxone, methylnaltrexone or alvimopan, are effective in reducing constipation without affecting pain relief and induce fewer withdrawal symptoms [169,170,171,172].

### 2.4. Nausea and Vomiting

Nearly 20% of patients under long-term opioid treatment experience nausea and vomiting [173]. The actual mechanism of opioid-induced nausea and vomiting is not clear, but the activation of opioid receptors (MOP or DOP) present in the chemoreceptor trigger zone, vestibular apparatus (MOP) and GI tract (MOP, DOP or KOP) is probably involved in the induction of nausea and vomiting [134]. At present, it is thought that these adverse effects are a direct consequence of opioid-induced effects in the area postrema of the brainstem, an area rich in dopamine, opioid and serotonin receptors [174,175]. In the clinic, 5-HT_3_ and NK_1_ receptor antagonists are used to prevent opioid-induced emesis, which could indicate that several non-opioid receptors (e.g., dopamine (D_2_), 5-HT_3_ and histamine (H_1_)) might interact with opioid receptors in those brain areas that control nausea and vomiting [134,176,177,178]. Although patients treated with oral morphine experience chronic nausea and vomiting, opioid rotation or changing the route of administration (e.g., oral to subcutaneous) appear helpful to reduce these adverse effects [168,179,180].

### 2.5. Respiratory Depression

**Figure 4:**
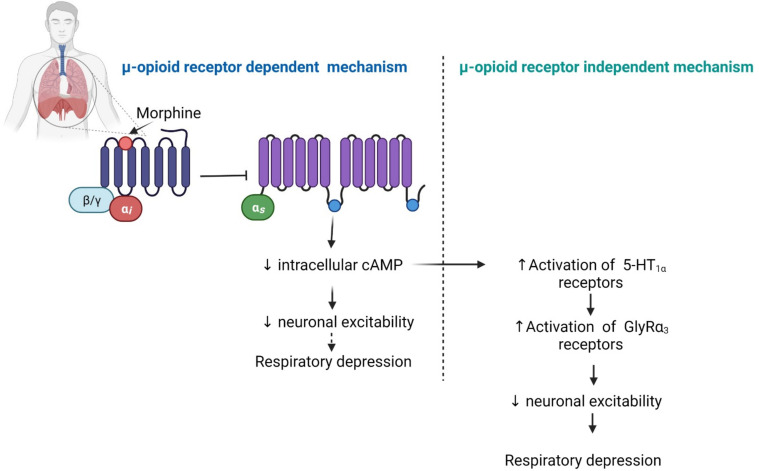
Effects of opioids on the respiratory function. Abbreviations: 5-HT, serotonin; GlyRα3, glycine receptor type-α3. Symbols: solid arrow, strongly connected; dashed arrow, moderately connected; upward arrow (symbol), increased effect; downward arrow (symbol), decreased effect. The figure was made with www.biorender.com (accessed on 19 October 2021).

Respiratory depression occurs less frequently compared to other adverse effects, but typically, it can have fatal consequences [149,181]. Similar to the other side effects, opioid-induced respiratory depression is mediated by the MOP receptor [182,183,184]. For example, fentanyl does not induce respiratory depression in MOP receptor knockout mice, indicating that the MOP receptor is responsible for respiratory depression [185]. Neurons of the pre-Bötzinger complex, a sub-region of the ventrolateral medulla, are responsible for controlling autonomic neuronal functions, including normal respiration [184]. The neurons of the pre-Bötzinger complex express a variety of receptors including neurokinin-1, serotonin (5-HT) and MOP receptors [184]. Inhibition of neurons that generate respiratory rhythms in the pre-Bötzinger complex cause respiratory depression [186] (Figure 4). MOP receptor activation inhibits adenylyl cyclase and reduces the synthesis of intracellular cAMP, which is thought to depress the respiratory neurons, as reduced cAMP levels in the cytoplasm reduces neuronal excitability by an unknown mechanism [134] (Figure 4). On the other hand, serotonin receptors in this region stimulate respiration [186,187]. The 5-HT1(a) receptors are expressed widely on respiratory neurons and are stimulated by reduced cAMP levels that activate the glycine receptor type α3 (GlyRα3) [188]. The activated GlyRα3 receptor inhibits neurons contributing to respiratory depression (Figure 4). This effect is independent of the MOP receptor-induced signal transduction pathway [188]. Therefore, multiple non-opioid receptors together with the MOP receptor are involved in the control of respiration and opioid-induced respiratory depression. Although high-dose opioid users are at risk of respiratory depression [189], a selective peripherally selective opioid antagonist can effectively reduce the incidence of respiratory depression without significant withdrawal symptoms [190].

### 2.6. Other Adverse Effects

In addition to analgesic tolerance, physical dependence and addiction as major adverse effects of long-term opioid treatment, this discussion also needs to address other behavioural side effects observed in the clinic [191]. Morphine-induced biphasic behavioural effects are well known from preclinical studies and include initial motor suppression and subsequent hyper-excitation [192,193,194,195,196,197,198]. An open-field arena is widely used to assess motor behaviour and typically includes horizontal movement, rearing (vertical movement) and turning behaviour. Morphine-induced horizontal locomotion, turning and circling behaviours are related to the dopaminergic system [195,199,200,201]. Morphine treatment induces the dopamine receptor-1 (D1)-dependent βarr-2/phospho-ERK (βarr2/pERK) signalling complex, which stimulates morphine-induced horizontal locomotion. However, the effects were absent in D1 and D2 receptor knockout mice [202]. Acute morphine administration induces phosphorylation of dopamine- and cAMP-regulated phosphoprotein of 32 kDa (DARPP-32), which activates the D1 receptor on dopaminergic neurons of NAc, substantia nigra and dorsal striatum and stimulates locomotor activity [202,203,204,205]. The D1, D2 and D3 receptors are responsible for the control of locomotion, learning and memory-related functions [206]. Opioids-induced activation of μ_1_-opioid receptor decreases gamma-aminobutyric (GABA) release in co-localised GABA_A_ receptors in the ventral tegmental area (VTA), but stimulates dopamine release in the NAc, as the dopaminergic neurons in the NAc emerge from the VTA [207] (Figure 2). The VTA, NAc (ventral striatum) and substantia nigra dopaminergic system is also involved in motivation-, reward- and addiction-related behaviour [208,209,210,211]. Noticeably, the effects of chronic morphine treatment on cAMP/PKA/DARPP-32 signalling are not fully understood at present. However, rearing behaviour can indicate increased exploration and reduced anxiety, which are related to GABA inhibitory neurotransmission [212,213,214]. Activation of the CREB transcription factor regulates anxiety-related behaviours, as CREB-deficient mice show an increased anxiogenic response [215]. However, the behavioural changes in response to chronic morphine treatment are independent of MOP receptor, cyclin-dependent kinase 5 (cdk5) or adenylyl cyclase activities in relevant areas of the brain [216]. Studies suggested that the morphine-induced behavioural effects are probably derived from its binding to the KOP receptor but not to the MOP receptor [217,218]. The likely multiple mechanisms that link chronic morphine treatment to its behavioural effects are not completely understood but may be controlled by a combination of dopaminergic, GABAergic, opioidergic and additional unknown neuronal signals [107,202,203,204,212,213,214]. The combination of multiple independent behavioural measurements is generally regarded as the most reliable approach to assess the total motor effects induced by opioids [193,194,219,220,221].

Drowsiness, lethargy, hyperalgesia and pruritus are also common adverse effects of opioids [222,223,224]. Suppression of motor behaviour causes drowsiness and lethargy; however, the exact mechanisms of this effect are not known. Morphine is responsible for generating an itching skin sensation by signalling through spinal heteromers of opioid and itch-mediating GPCRs [225,226]. Withdrawal after chronic morphine exposure also induces histamine-induced itching or scratching responses [227]. Furthermore, morphine can induce increased pain sensation (hyperalgesia) via the MOP receptor [228]. However, the molecular mechanism of morphine-induced hyperalgesia is not well understood but might be related to the upregulation of protein kinase C gamma (PKCγ) and the *N*-methyl-*D*-aspartate (NMDA) receptor subtype NR1 in the spinal cord [229,230]. It is also thought that different MOP receptor isoforms, functional interactions with other GPCRs or opioid metabolites, such as morphine 3-glucuronide that interacts with GABA or NMDA receptors, could be responsible for this adverse effect [224,228,231].

## 3. Discussion and Future Directions

Activation of one opioid receptor type might likely affect the behaviour of another opioid receptor type in the same complex, such as in the form of receptor homodimers or heterodimers. Different interactions exist between two or multiple receptors [70,232,233,234], which depend on the pharmacological profile of the ligands that interact with these receptors [235,236]. The current literature suggests that most opioid receptor ligands are not extremely selective and could therefore bind to one/more off-target receptors to produce beneficial/therapeutic effects and unwanted adverse effects. For example, morphine at high doses can elicit analgesia in MOP receptor knockout mice by activating the KOP receptor [237]. Similarly, in DOP receptor knockout mice, a DOP receptor agonist effectively produces analgesia, while a non-specific opioid antagonist (naltrexone) could reverse this effect [68]. Although these data could be interpreted as non-specific interactions of the different DOP receptor agonists and antagonists, they have been interpreted as evidence of the presence of a different DOP receptor subtype [68]. However, recent studies suggest that opioid receptor subtypes may not actually exist, but these results rather reflect the presence of homo- or heteromeric receptor dimers [14]. In light of a reduced DOP receptor activity in MOP receptor knockout mice [70], it was hypothesised that the specific interaction between MOP and DOP receptors in specific neural pathways could modulate pain perception. In line with this hypothesis, co-administration of morphine and a DOP receptor antagonist induces analgesia, while surprisingly reducing tolerance in rodent models [130,131,132,133,238,239], which suggests that MOP and DOP receptor interactions regulate antinociceptive tolerance. It has to be noted that respiratory depression was not prevented under these conditions, which suggests that additional receptor interactions are likely to be involved [132].

These studies justify the approach to target two opioid receptors simultaneously to explore the molecular mechanisms that contribute to tolerance and develop alternative drug candidates with reduced risk for the development of tolerance. The challenge is to develop multi-target-specific ligands that are effective as analgesics, with a favourable side effect profile. Several strategies for the simultaneous targeting of multiple receptors can be envisaged: (i) co-administration of two selective drugs, (ii) administration of one non-receptor-selective drug or (iii) use of a single drug that specifically targets different receptors (i.e., multiple-receptors-selective ligand) [240]. Particularly, the third strategy promises clinical advantages by reducing drug–drug interactions, as well as by allowing pharmacokinetics and pharmacodynamics that will be easier to control [240,241,242].

Various new opioid receptor ligands have been designed to target two or more opioid receptor types simultaneously, and many of these are effective in producing an analgesic response in vivo. Rational drug design and structure–activity relationship studies have evaluated the pharmacology of many of those ligands that act simultaneously on two or more different opioid receptors [242,243,244,245,246] or a combination of an opioid receptor with a non-opioid receptor [247,248,249]. For example, MDAN-21 is a mixed MOP receptor agonist/DOP receptor antagonist that is 50-fold more potent than morphine and produces less tolerance [32]. This effect is likely the consequence of reduced internalisation of MOP–DOP receptor heterodimers due to the bridging of the two receptors [250]. Another recent example is KGFF09, a bifunctional G-protein-biased MOP agonist–neuropeptide FF antagonist effective against acute nociceptive and inflammatory pain and with improved acute and chronic side effects [251]. The association of the two properties within a single molecule gathers the beneficial effects of G-protein-biased MOP receptor agonism on acute side effects (respiratory depression) and those of neuropeptide FF receptor antagonism on chronic side effects (opioid-induced hyperalgesia, analgesic tolerance and withdrawal syndrome). The most advanced among bifunctional opioid ligands is cebranopadol, a MOP/NOP agonist in advanced clinical development for the treatment of acute and chronic pain [252].

Studies showed that the chronic administration of morphine increased the expression of MOP–DOP receptor heteromers in the rostral ventral medulla in the brainstem, which is responsible for the processing of nociceptive responses [142,253]. Different opioid ligands have been focused on targeting the MOP–DOP receptor dimers with the objective that the co-expression of the MOP and DOP receptors might reduce analgesic tolerance [142,254,255,256]. Some of these ligands showed selectivity for the MOP–DOP receptor dimer, as well as for the individual MOP or DOP receptors [254], but other ligands did not show selectivity towards the individual receptors [256]. MOP–DOP receptor heteromer-biased ligands can activate both opioid-mediated and β-arr2-mediated signalling. An anti-analgesic effect of the MOP–DOP heteromers was also observed [257]. The adverse effects profile of heteromer-selective ligands are not clearly known [253], but research related to opioids with selectivity for heteromers and/or individual receptors is advancing.

Altogether, all clinical available and experimental opioids have some sort of adverse effects, but their clinical application should be based on the needs of individual patients and on the measurement of the risk/benefit ratio of a particular drug. Additionally, the complexity of chronic pain syndromes requires tailored pharmacological interventions and innovative drugs to effectively and safety control pain. Several experimental drugs (e.g., opioids, ion channel inhibitors) can be administered in combination (if they prove to be safe) with clinical opioids (e.g., intrathecal ziconotide, an N-type calcium ion channel inhibitor, ω-conotoxins with intrathecal morphine) and can provide better pain relief and less adverse events than opioids alone in clinical trials [258,259]. Further extensive work on the efficacy of new opioids or combination therapies is necessary to manage opioid-related adverse effects in clinical settings.

## Figures and Tables

**Table 1:** Major types of opioid receptors.

Receptor Nomenclature ^1^	Gene	Most Common Location in the CNS ^2^	Most Common Roles and Functions	Selective Agonist ^3^	Selective Antagonist ^4^
μ, mu, MOP	OPRM1	Thalamus, amygdala, dorsal horn, cerebral cortex, striatum, hippocampus, locus coeruleus	Analgesia, intestinal transit, feeding, mood, hormone secretion, thermoregulation, cardiovascular function	DAMGO, sufentanil, PL017	CTAP, CTOP, β-FNA
δ, delta, DOP	OPRD1	Olfactory bulb, thalamus, cortex, caudate putamen, nucleus accumbens (NAc), amygdala, dorsal horn	Analgesia, mood, gastrointestinal motility, behaviour, cardiovascular regulation	DPDPE, [D-Ala^2^]deltorphin I, [D-Ala^2^]deltorphin II SNC80	Naltrindole, TIPPᴪ, Naltriben
κ, kappa, KOP	OPRK1	Olfactory bulb, NAc, cerebral cortex, claustrum, amygdala, caudate nucleus, hypothalamus, subthalamic nucleus, thalamus, corpus callosum.	Analgesia in inflammation, diuresis, feeding, neuroprotection, neuroendocrine functions	Enadoline, U50488, U69593, salvinorin A	norBNI, GNTI
N/OFQ, NOP	OPRL1	Hippocampus, hypothalamus, amygdala, substantia nigra, dorsal horn, lateral septum	Spinal analgesia, anxiety, mood, memory, feeding, locomotor activity	UFP-102, Ro64-6198, N/OFQ-(1-13)-NH_2_, UFP-112	UFP-101, SB 612111, J-113397, JTC-801

^1,2,3,4^ Further detailed information can be obtained from IUPHAR/BPS guide to pharmacology (http://www.guidetopharmacology.org; accessed on: 26 October 2021) and Alexander and colleagues [14]. Abbreviations: β-funaltrexamine (β-FNA), [D-Ala^2^, *N*-MePhe^4^, Gly-ol]-enkephalin (DAMGO), norbinaltorphimine (norBNI), [D-Pen^2^, D-Pen^5^] enkephalin (DPDPE), nociceptin/orphanin-FQ (N/OFQ), H-Tyr–Tic–Phe–Phe-OH (TIPP).

## Data Availability

Not applicable.
